# Low Infrared Emissivity Coating Based on Graphene Surface-Modified Flaky Aluminum

**DOI:** 10.3390/ma11091502

**Published:** 2018-08-22

**Authors:** Lihua He, Yan Zhao, Liying Xing, Pinggui Liu, Youwei Zhang, Zhiyong Wang

**Affiliations:** 1 School of Materials Science and Engineering, Beihang University, Beijing 100191, China; 2 Beijing Institute of Aeronautical Materials, Beijing 100095, China; vcd4321@sina.com (L.X.); liupinggui@hotmail.com (P.L.); ywzhang_pku@163.com (Y.Z.); zywang910@163.com (Z.W.)

**Keywords:** graphene, flaky aluminum powder, low infrared emissivity coating, glossiness, anticorrosive performance

## Abstract

A low infrared emissivity coating was prepared using graphene surface-modified flaky aluminum complex powders (rGO-FAl) as fillers. The flaky aluminum powders were coated with graphene through chemical bonding. Compared with pure flaky aluminum, the Vis-NIR diffuse reflectance of rGO-FAl complex powders was significantly decreased, which was beneficial to the low glossiness of the coating. After the modification, the glossiness at 60° of the coating with 40% (mass fraction) pigments decreased from 12.8 to 6.7, while the coating maintained low infrared emissivity (0.238~0.247) at a spectral range of 8–14 μm. In the electrochemical impedance spectroscopy (EIS) measurement, at the lowest frequency, the impedance of the Al-rGO test plate was at least two orders of magnitude greater than that of the control Al test plate, and the graphene layer significantly increased the bandwidth of the maximum phase angle, which indicates a good protective effect of the ultra-thin graphene layer on metal in a corrosive environment. The coating with 40% rGO-FAl complex powders can maintain its appearance after 500 h of salt spray corrosion testing. In contrast, the color of the coating with the original aluminum powders changed after only 300 h.

## 1. Introduction

Aluminum has been widely applied in industry due to its excellent electrical and thermal conductivity. Additionally, aluminum particles are also used as main filler in low infrared emissivity coatings [1]. Numerous works have focused on low infrared emissivity coatings using flaky aluminum powders as the fillers [2,3]. The influence of content, diameter of fillers and thickness of coating on infrared emissivity has been systematically studied. It is clear that the high volume content of aluminum powders is necessary to achieve low infrared emissivity of coatings. However, the high content of aluminum powders usually leads to high glossiness, and bad anticorrosive and mechanical properties of coating.

Given that the low infrared emissivity coating always represents a physical barrier between the substrate and the environment, the anticorrosive performance of coating is very crucial. Furthermore, high glossiness also constrains the application of aluminum powder [2], which can result in bad compatibility of visible and near-infrared light, especially in military applications. Anticorrosion paints often consist of organic binder and anticorrosive pigments dispersed in binder. The anticorrosive pigments are also needed to obtain corrosion-inhibiting properties. Chromate is the most common prominent anticorrosive pigment. However, its toxicity and harmfulness to the environment limit its application. Thus, it is normally replaced by phosphate, molybdate, borate or vanadate (IV) [4,5,6,7]. Nevertheless, the infrared emissivity of the coating will be increased when the anticorrosive pigments are added. Therefore, a highly effective strategy for designing low infrared emissivity coating with favorable anticorrosive property is urgent in this field.

Recently, the glossiness of low infrared emissivity coatings was reduced by adding carbon black into aluminum powders [2,3,8], leading to an increase in infrared emissivity because of the high infrared emissivity characteristics of carbon black. Graphenes have attracted much attention due to their unique features, such as high thermal and electrical conductivity, high strength, and chemical stability [9,10,11,12,13]. Graphenes are also promising in anticorrosion coatings due to their good anticorrosive performance [14,15,16,17]. It is noteworthy that structures comprised of graphene with other particles enhance coating properties better than pure graphene [18,19,20,21,22,23]. Since the energy gap of graphene is zero, graphene will not generate thermal radiation, according to the principles of thermal radiation theory. These characteristics further reduce the infrared emission rate of graphenes.

If aluminum particles are coated with graphene, the complex will exhibit both good anticorrosive property and low infrared emissivity and glossiness. However, methods for designing the graphene/aluminum complex to achieve low infrared emissivity, glossiness and high anticorrosive properties represent an emerging field. In this work, graphene surface-modified flaky aluminum powders and their coatings were prepared. In addition, the visible light to near-infrared (Vis-NIR) diffuse reflection characteristics, as well as glossiness and anticorrosive properties, were studied.

## 2. Experimental

### 2.1. Materials

Natural graphite powders were purchased from Qing Dao Tian Sheng Co. Ltd. (Qingdao, China) with an average diameter of 48 μm. Flaky aluminum powders (FAl) were purchased from Beijing Chemical Factory (Beijing, China). The 3-aminoproplyphosphoic acid (APPSA) was obtained from TCI (Shanghai, China). Tetrahydrofuran ether polyurethane prepolymer (PTMG-PU) was supplied by Shandong Dong Da Yi Nuo Wei Polyurethane Corporation (Zibo, China). The polyaspartic ester (NH1420) used as crosslinker was obtained from Bayer (Pittsburgh, PA, USA). The content of non-volatile components in used the polyurethane resin and crosslinker were all 100 wt.%. Ethanol (purity: ≥99.7%) and xylene (purity: ≥99.0%) were purchased from Beijing Chemical Factory (Beijing, China) and were used without further purification.

### 2.2. Preparation of Graphene Surface-Modified Flaky Aluminum Powders (rGO-FAl)

The graphene oxides (GO) were prepared from natural graphites by a modified Hummers method [24]. The as-prepared GO were first dispersed in deionized water to form a 1 mg mL^−1^ suspension by ultrasonication for 2 h. Subsequently, a certain amount of APPSA was added. The mixture was refluxed at 95 °C for 20 h, then dialyzed with cellulose ester dialysis membrane (MWCO is 500~1000 Da) in deionized water for 7 days to remove the unreacted APPSA. The mixture was dried in a vacuum oven at 60 °C for 24 h and the -PO(OH)_2_ functionalized GO (GO-APSA) was prepared. The FAl powders were washed by ethanol before dispersing in a mixture of ethanal/H_2_O (9:1, *v*/*v*). A certain amount of GO- APSA was dispersed in water by ultrasonication and added in the suspension of FAl powders. The mixture was then heated to 40 °C and stirred for 4 h. The liquid mixture was then filtered and washed with ethanal/H_2_O (9:1, *v*/*v*) to remove the unreacted GO-APSA. The filter cake was heated at 150 °C in a vacuum oven for 24 h to obtain rGO-FAl powders. Different contents of rGO in rGO-FAl powders (1% rGO-FAl, 2% rGO-FAl, 5% rGO-FAl) were produced by changing the amount of the GO-APSA in the reaction between the GO-APSA and the FAl powders. At the same time, the control composite of 5% rGO/Al, was prepared by ultrasonic mixing of GO nanosheets and FAl with the same chemical composition. The formulations of 1% rGO-FAl, 2% rGO-FAl, 5% rGO-FAl and 5% rGO/FAl powders are listed in Table 1.

### 2.3. Preparation of Low Infrared Emissivity Coating

The clean aluminum (Al) substrate (10 cm × 10 cm, thickness 2 mm) was used as the coating substrate to measure the infrared emissivity, glossiness and anticorrosive properties. First, a fixed amount of PTMG-PU, xylene and the filler were mixed and dispersed using a high-speed dispersion machine (FA25, FLUKO, Shanghai, China) at 10,000 rpm for 5 min. Then a certain amount of the crosslinker was added and mixed by hand. The viscosity of the paint was adjusted by xylene prior to spray. Finally, the paint was sprayed (SATA, Stuttgart, Germany) on the Al substrate with accurate speed and pressure. After drying at 60 °C for 24 h, the coating was prepared at a thickness of 40~50 μm. The mass fraction of the fillers were all 40%. The formulations of the coatings are listed in Table 2.

### 2.4. Characterization

The morphology of the rGO-FAl and FAl particles was analyzed by field emission scanning electron microscopy (Quanta 2005, FEI, Hillsboro, AL, USA). In addition, the samples were mounted on aluminum studs using adhesive graphite tape and sputter-coated with gold before analysis.

Raman spectra were recorded on a confocal Raman spectrometer system (Renishaw, In Via) using a 532 nm laser as the excitation source. Diffuse reflectance Vis-NIR spectra was measured by using UV/VIS/NIR spectrometer (Lambda 750, Perkin-Elmer, Waltham, MA, USA) using a 150 nm polytetrafluoroethylene integrating sphere.

Electrochemical impedance spectra (EIS) measurements were carried out in 3.5% NaCl solution at room temperature with a PARSTAT 2273 electrochemical system (Princeton Applied Research, Princeton, NJ, USA). EIS is a technique with a signal that only has small perturbations, and the surface damage of the sample is very low. EIS were obtained at open circuit potential with a 10 mv sine perturbation. The measuring frequency range was 10^−2^~10^5^ Hz. The exposed area of the working electrode was about 1 cm^2^. The reference electrode was saturated silver chloride electrode (SCE). A graphite electrode was used as a counter electrode. ZsimpWin software was used to analyze the electrochemical impedance spectra in order to study the corrosion process of graphene coating.

The coating thickness was measured using a digital magnetic thickness instrument (Qingdao, China), with a standard instrumental error of ±1 μm. The infrared emissivity values of the samples within 8~14 μm were measured by SR-5000 infrared emissometer (Shanghai Institute of Technological Physics, Shanghai, China). A WGG-type glossinessmeter was used to measure the surface glossiness of the coating, at an incidence angle of 60°. The corrosion behavior of the coatings was measured by a 500 h salt spray test. The test was performed by exposing the sample in a testing chamber, and was carried out using a continuously sprayed mist of 5% aqueous solution of sodium chloride at 35 °C as corrosive medium.

## 3. Results and Discussion

### 3.1. Characterization of rGO-FAl Complex Particles

**Figure 1:**
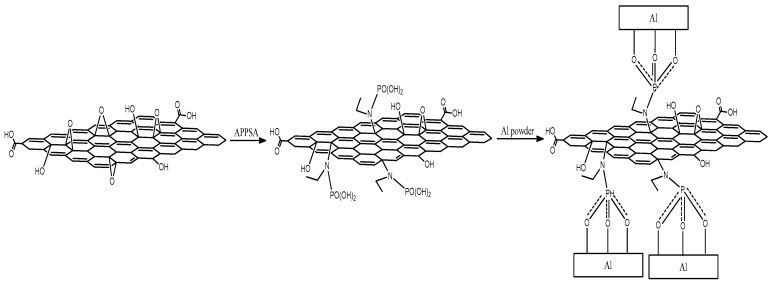
The schematic route of the preparation of rGO-FAl complex particles.

The preparation procedure of the rGO-FAl complex particles is illustrated in Figure 1. Using APSA as a “link” agent, the rGO nanosheets can be tightly coated onto the surface of FAl. In detail, the GO, prepared by the Hummers method, were firstly functionalized with -PO(OH)_2_ by the reaction of amine groups of APSA and the carboxyl groups of GO [25]. The remaining -PO(OH)_2_ functional groups on GO were then reacted with FAl to obtain GO-modified FAl powders. Finally, the GO-modified FAl particles were transformed in situ into rGO-FAl complex particles through thermal reduction treatment of GO [25].

**Figure 2:**
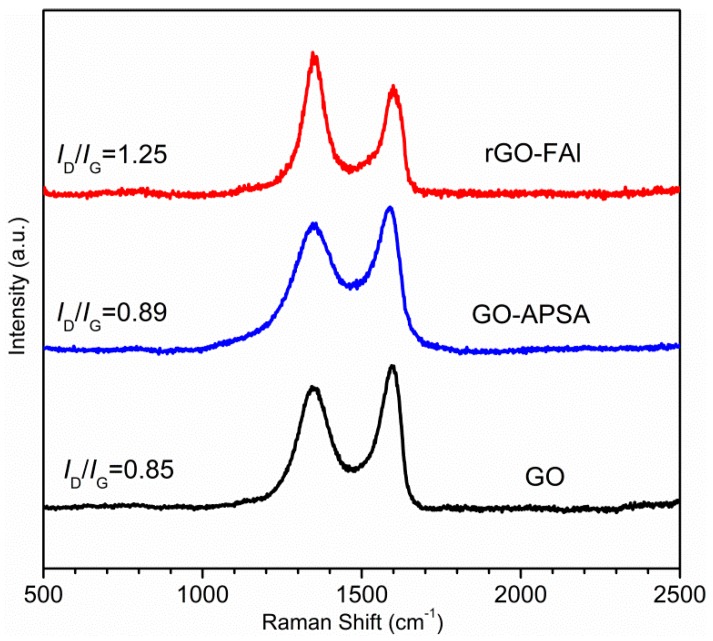
Raman spectra of GO, GO-APSA, and rGO-FAl complex particles.

In our previous work [25], the functionalization of GO with APSA was characterized and analyzed by FT-IR spectra, XRD, XPS and TEM. Here, we mainly focus on the analysis of rGO-FAl powders. It is known that the bonding state of carbon atoms in carbon materials can be described by Raman spectra, and thus it can be used to analyze the evolution of carbon-based materials during the synthesis process. Figure 2 shows the Raman spectra of GO, GO-APSA, and rGO-FAl powders. The three samples all show two distinguishable peaks assigned to D- and G-bands at 1344 cm^−1^ and 1580 cm^−1^, respectively. Their ID/IG value is different. According to the exact interpretation proposed by Ferrari and Robertson [26], the D-band is a breathing mode of A_1g_ symmetry involving phonons near the K zone boundary, which is forbidden in perfect graphite and becomes active in the presence of disorder or finite-size crystals for graphite (nanographite crystals). The G-band corresponds to the E2g mode due to stretching vibrations of sp^2^ bond. The ratio of D and G bands, ID/IG, is useful to evaluate the structural disorder of graphene nanosheets. After the functionalization of GO with APSA, the value of ID/IG increases slightly, from 0.85 for GO to 0.89 for GO-APSA, and the slightly higher intensity of the D band suggests the presence of a bit more disorder in GO-APSA due to the bonding effect between amine groups of APSA and the carboxyl groups of GO. The value of ID/IG increases significantly from GO-APSA to rGO-Al (from 0.89 to 1.25), reflecting the increase in disorder, which may be derived from the reaction between the -PO(OH)_2_ groups of GO-APSA and the FAl powder surface.

**Figure 3:**
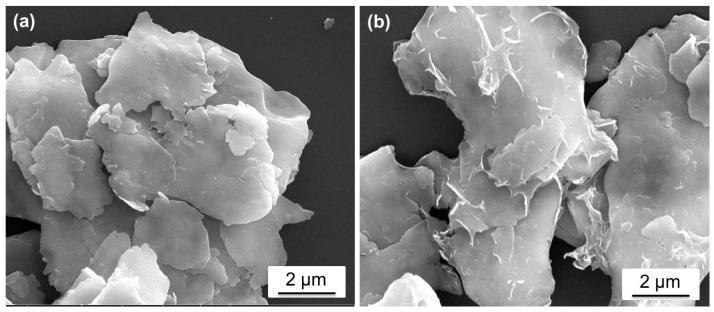
FE-SEM images of aluminum particles (**a**) and rGO-FAl complex particles (**b**).

The morphology of the as-prepared rGO-FAl complex particles and FAl powders were observed by FE-SEM (Figure 3). The surface of the FAl (Figure 3a) was smooth and clean, whereas the rGO-FAl (Figure 3b) was rough and covered with graphene nanosheets. The surface of the FAl was smooth, but the rGO-FAl complex particles showed a rough surface. This can be attributed to the presence of the graphene and the reaction between the FAl and the -PO(OH)_2_.

### 3.2. The Vis-NIR Diffuse Reflection Characteristics of rGO-FAl Powders

**Figure 4:**
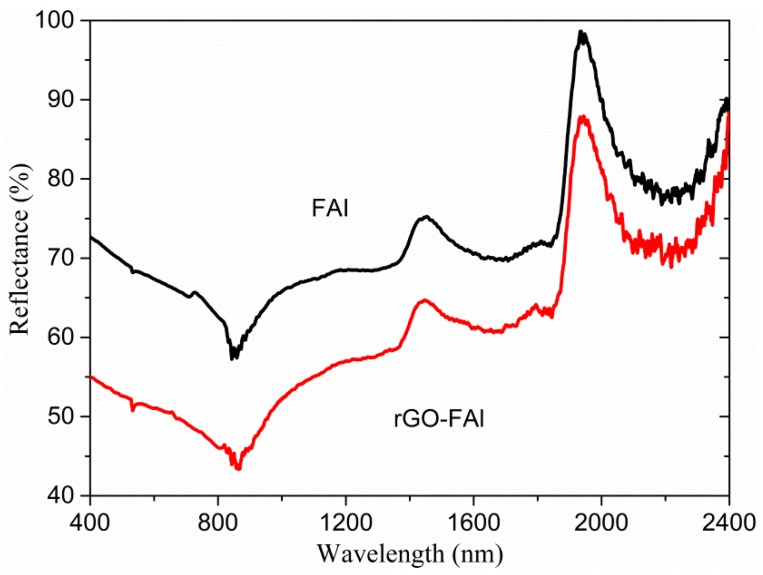
Diffuse reflectance Vis-NIR spectra of flaky Al and rGO-FAl powders.

The glossiness of the coating was directly affected by the Vis-NIR diffuse reflection of fillers. Figure 4 shows the visible light to near-infrared diffuse reflection spectra of the FAl powders and the rGO-FAl powders. Although an obvious characteristic absorption peak appears near 840 nm, the FAl powders show a very high spectral reflectance at visible light and near-infrared wavelengths. The spectral reflectance of rGO-FAl powders was significantly decreased. The average reflectance of rGO-FAl powders at 400–760 nm, 760–2400 nm dropped by 16.7% and 10.3%, respectively, in comparison to FAl powders. The characteristic fold structure of rGO-FAl powders increases the surface roughness. Based on Figure 3, the roughness of the rGO-FAl particle is in nanometer scale, similar to the wavelength of visible light. When visible light reaches the surface of the composite powders, the rough surface will cause strong multi-correlation scattering and absorption [27,28]. In addition, the absorption path of radiation in the filler system is increased; thus, the filler has the effect of extinction and the spectral reflectance is decreased.

### 3.3. Infrared Emissivity and Glossiness of rGO-FAl Coating

To study the influence of FAl powers and rGO-FAl complex powders on the glossiness and emissivity of the coatings, experiments were carried out using the same processing. The mass fractions of the fillers in the coatings were all 40%. The results of infrared emissivity within 8~14 μm and glossiness at 60° are summarized in Table 1. The influence of graphenes was further investigated by a series of separate experiments, in which the mass fraction of rGO-FAl powder was fixed at 40%. The ratio of graphene in rGO-FAl was varied between 1%, 2% and 5%, respectively. From Table 3, the coating glossiness with FAl powders is much higher than that with rGO-FAl powders. The specular glossiness of a coating film should not be high. It should especially not be more than 10 when the angle is 60°. It was found that the content of graphene in rGO-FAl had a strong effect on the glossiness of the coating. When the ratio of rGO in rGO-coated Al was increased from 0% to 5%, the glossiness of the coating decreased from 12.8 to 6.7. This can be attributed to the rough surface after the FAl powers were coated with rGO. In terms of the infrared emissivity of the coating, the infrared emissivity of the coating with FAl powders was 0.258, while the infrared emissivity of the coating, with various content of rGO in the rGO-FAl complex powders, was 0.238~0.247. It can be seen that the infrared emissivities of the FAl powders and the rGO-FAl complex powders were similar, meaning that graphenes are not able to increase the infrared emissivity.

Based on Kirchhoff’s law, when a system is isothermal and in thermal equilibrium, the absorbance is strictly equal to the emissivity [3]. Because the energy gap of graphenes is zero, according to the principle of thermal radiation generation, graphenes themselves will not generate thermal radiation. These characteristics further reduce the infrared emission rate of graphenes. Therefore, using rGO-FAl powders as the fillers of the low infrared emissivity coating, the coating glossiness will be dramatically decreased, while the emissivity can remain very low.

To verify this, a control composite of rGO/Al was prepared by ultrasonic mixing of GO nanosheets and flaky Al with the same chemical composition. The control composite displays a completely different microstructure from rGO-FAl, in which the rGO nanosheets are aggregated together and the external surface of FAl is covered by nothing (Figure S1). Although the rGO/Al coating (Table 3) showed the least glossiness, the value of infrared emissivity at 8~14 μm is higher than those of the FAl and rGO-FAl coatings. The reason for this is that the agglomeration of rGO particles (~20 μm) increases the roughness of the coating, leading to a significant absorption increase and reflectance decrease of the electromagnetic wave. Therefore, compared to the smooth FAl and rGO-FAl coatings, the rough rGO/Al coating shows higher emissivity.

### 3.4. Anticorrosive Performance

In our previous work [25], it was proved that GO attached onto aluminum powder could effectively improve the anticorrosive performance of the pigments. The electrochemical impedance technique was employed to evaluate and compare the corrosion resistance of the composite coating. To further confirm the effect of graphene coating on metal corrosion resistance, an electrochemical test was carried out. Compared to the hydrogen evolution experiment, the flaky aluminum powders were replaced by Al test plates (2 cm × 2 cm, thickness 2 mm). The Al test plates were placed in the GO solution functionalized by phosphonic acids, and the graphene oxide layer was formed on the surface of the Al test plate by a chemical reaction between phosphonic acids and metals. Then, the graphene film coating Al test plate (Al-rGO) was obtained by washing and dried in a vacuum oven at 150 °C for 24 h. At the same time, a control aluminum test plate (Al) was compared. The Al test plate was placed in non-functional GO, and the rest of the washing and thermal treatment processes were similar. By characterizing the electrochemical curve of Al-rGO test plates and the control Al test plate, the effect of the graphenes themselves on improving the corrosion resistance of the test plate was obtained. When using electrochemical impedance spectroscopy (EIS) to understand the electrochemical degradation of metals and their coated samples, Bode plots are the most common representations of the processes occurring at various interfaces. In Bode plots, the magnitude of the impedance (Z) and phase-angle are plotted respectively as a function of frequency (ω). In the case of the Bode plots, the magnitude of the impedance at the lowest frequency also represents the corrosion resistance [29,30].

**Figure 5:**
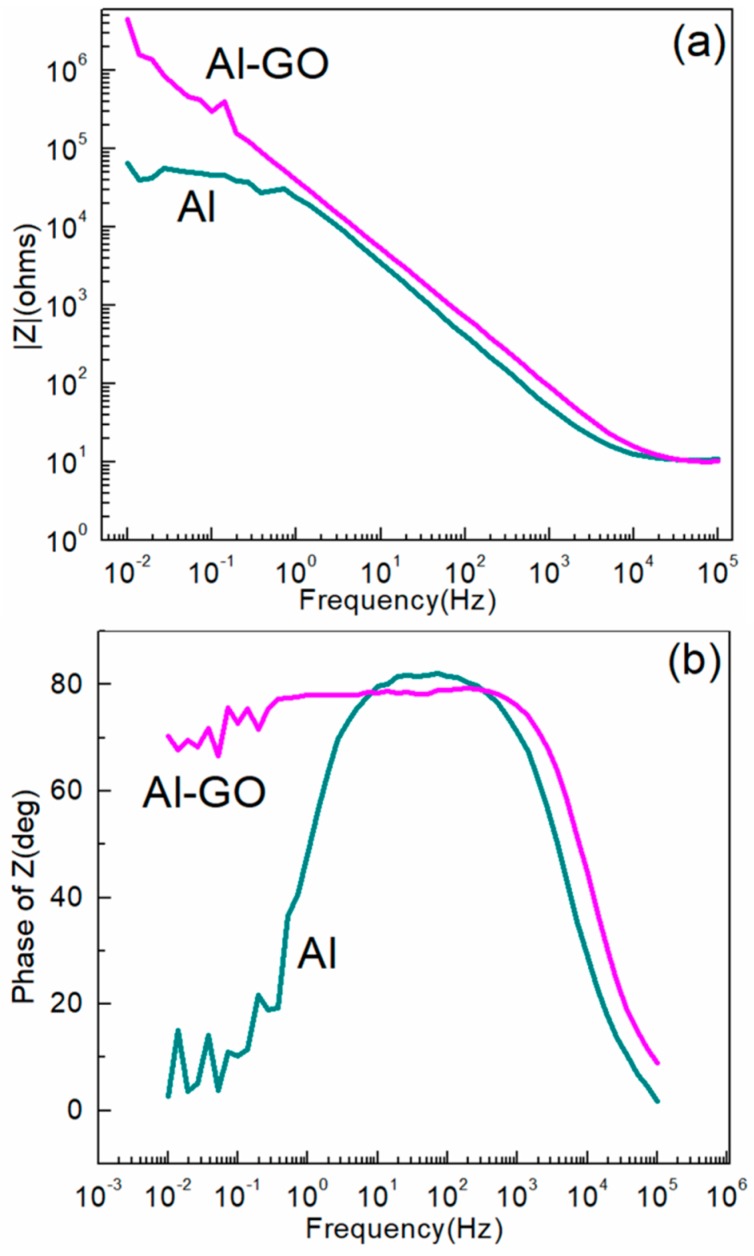
Bode plots of the rGO-coated Al test plate and the control Al test plate: modulus (**a**) and phase angle (**b**) Plots.

According to the analysis of impedance modulus at low and medium frequencies in Figure 5a, at the lowest frequency, the impedance of Al-rGO test plate is at least two orders of magnitude greater than that of the control Al test plate. This indicates that the corrosion resistance of the Al-rGO test plate is obviously better than that of the control Al test plate. In preparing the control Al test plate, there was no graphene bonded onto the surface of the Al plate. Even if GO were physically attached to the surface of the Al plate, the graphene oxide would be washed away in subsequent washing due to the absence of chemical bonds. However, the surface of the Al-rGO test plate is firmly attached to graphene layer due to the chemical reaction between -PO(OH)_2_ and Al. The graphene membrane can be impermeable to standard gases, including helium, because of its highly efficient stratified barrier [31,32,33,34,35]. Additionally, graphene has a hydrophobic nature due to its non-polar covalent double bonds, which prevent hydrogen bonding with water [36]. From the phase angle plots (Figure 5b), the presence of the graphene layer significantly increases the bandwidth of the maximum phase angle, which also proves the good protective effect of the pure graphene layer on the metal in corrosive environments.

**Figure 6:**
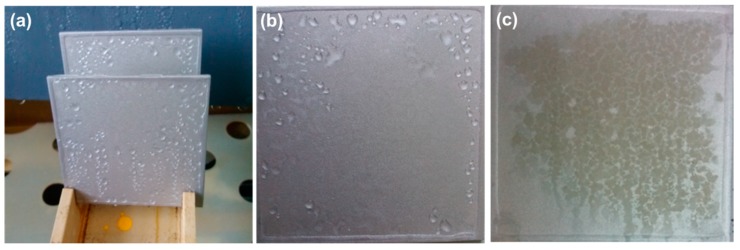
The picture of coating salt spray testing: (**a**) before the salt spray test, (**b**) the coating with rGO-FAl powder after 500 h salt spray testing, and (**c**) the coating with FAl powder after 300 h salt spray testing.

To compare the anticorrosive performance of the coating, the samples of the FAl powder and rGO-FAl powder coatings for testing infrared emissivity were measured under 500 h salt spray testing. Figure 6a is the picture of placing the two samples in the testing chamber. Figure 6b,c shows the results of the salt spray test. The coating with rGO-FAl powder maintained its appearance after 500 h of salt spray corrosion testing, while the color of the sample coated with the FAl powder changed in only 300 h. This contrasting property is attributed to the structure of the graphene films, which consist of millions of small flakes stacked randomly on top of each other. Water molecules and gases were prevented from penetrating the coating because of the highly efficient stratified barrier and the hydrophobic nature of graphene. On the other hand, the graphene, acting as a barrier for protecting the aluminum from corrosion/oxidation, can firmly attach to the surface of the aluminum flake by chemical bonds, and would exhibit a good dispersion in polymer coatings, which is favorable for enhancing the anticorrosive properties of coatings.

## 4. Conclusions

A low infrared emissivity coating was prepared using graphene surface-modified flaky aluminum complex powders (rGO-FAl) as pigments. After the aluminum powders were coated with graphene through chemical bonding, the glossiness at 60° of the coating with 40% pigment decreased from 12.8 to 6.7. The coating maintained its low infrared emissivity (0.238~0.247) at a spectral range of 8–14 μm. The good protective effect of the ultra-thin graphene layer on metal can be achieved in corrosive environmenta. The coating with 40% rGO-FAl powders can maintain its appearance after 500 h of salt spray corrosion testing. In contrast, the color of the coating with the FAl powders changed after only 300 h. It is expected that a coating using rGO-FAl complex powders would exhibit low infrared emissivity, low glossiness and good anticorrosive performance.

## Figures and Tables

**Table 1:** The formulations of 1% rGO-FAl, 2% rGO-FAl, 5% rGO-FAl and 5% rGO/FAl powders.

Content of rGO	GO-APSA (g)	GO (g)	FAl (g)	Color
1% rGO-FAl powder	0.1	-	10	Silvery white
2% rGO-FAl powder	0.2	-	10	Silvery white
5% rGO-FAl powder	0.5	-	10	Silvery white
5% rGO/FAl powder	-	0.1	10	Silvery white

**Table 2:** The formulations of the coatings.

No.	PTMG-PU (wt. Part)	Crosslinker (wt. Part)	FAl (wt. Part)	1% rGO-FAl (wt. Part)	2% rGO-FAl (wt. Part)	5% rGO-FAl (wt. Part)	5% rGO/FAl (wt. Part)
1	47	13	40	-	-	-	-
2	47	13	-	40	-	-	-
3	47	13	-	-	40	-	-
4	47	13	-	-	-	40	-
5	47	13	-	-	-	-	40

**Table 3:** The infrared emissivity and surface glossiness of coatings with 40% mass fraction of fillers.

No.	Filler Type	Infrared Emissivity (8~14 μm)	Surface Glossiness (60°)
1	Pure flaky Al powder	0.258	12.8
2	1% rGO-FAl powder	0.247	9.7
3	2% rGO-FAl powder	0.238	8.8
4	5% rGO-FAl powder	0.243	6.7
5	5% rGO-FAl powder (the control composite)	0.315	6.7

## Supplementary Materials

The following are available online at http://www.mdpi.com/1996-1944/11/9/1502/s1. Figure S1: SEM image of control composite of rGO/Al powders.
