# Supplementary material for: Low Infrared Emissivity Coating Based on Graphene Surface-Modified Flaky Aluminum
Source: Materials (Basel). 2018 Aug 22;11(9):1502. doi: 10.3390/ma11091502 (PMC6163430; doi:10.3390/ma11091502)
Supplement: Supplementary file 1 [file materials-11-01502-s001.pdf]

Supplementary Materials

Low Infrared Emissivity Coating Based on Graphene Surface-Modified Flaky Aluminum

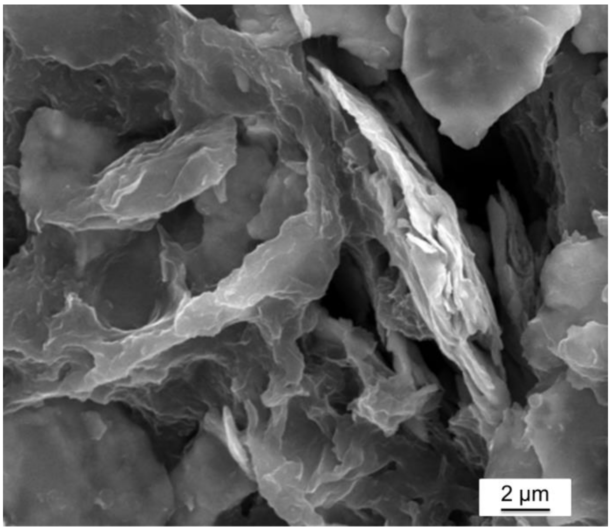

Figure S1. SEM image of control composite of rGO/Al powders.
